# Degradation of Triazole Fungicides by Plant Growth-Promoting Bacteria from Contaminated Agricultural Soil

**DOI:** 10.4014/jmb.2308.08037

**Published:** 2023-10-26

**Authors:** Thi Tham Luong, Thi Hong Tham Nguyen, Tien Dat Nguyen, Van Toan Le, Thi Hong Trang Pham, Thanh-Tam Ho, Ngoc-Loi Nguyen

**Affiliations:** 1 Da Lat Nuclear Research Institute, 01 Nguyen Tu Luc, Da Lat 66106, Lam Dong, Vietnam; 2 Institute for Global Health Innovations, Duy Tan University, Da Nang 550000, Vietnam; 3 Biotechnology Department, College of Medicine and Pharmacy, Duy Tan University, Da Nang 550000, Vietnam; 4 Institute of Oceanology, Polish Academy of Sciences, Powstancow Warszawy 55, Sopot 81-712, Poland

**Keywords:** Triazole fungicides, biodegradation, plant growth promotion, soil microorganisms, microbial consortium

## Abstract

The widespread application of triazole fungicides (TFs) in agricultural practices can result in the considerable accumulation of active compound residues in the soil and a subsequent negative impact on the soil microbiota and crop health. In this study, we isolated three TF-degrading bacterial strains from contaminated agricultural soils and identified them as *Klebsiella* sp., *Pseudomonas* sp., and *Citrobacter* sp. based on analysis of morphological characteristics and 16S rRNA gene sequences. The strains used three common TFs, namely hexaconazole, difenoconazole, and propiconazole, as their only sources of carbon and energy for growth in a liquid mineral salt medium, with high concentrations (~ 500 mg/l) of each TF. In addition to the ability to degrade fungicides, the isolates also exhibited plant growth-promoting characteristics, such as nitrogen fixation, indole acetic acid production, phosphate dissolution, and cellulose degradation. The synergistic combination of three bacterial isolates significantly improved plant growth and development with an increased survival rate (57%), and achieved TF degradation ranging from 85.83 to 96.59% at a concentration of approximately 50 mg/kg of each TF within 45 days in the soil-plant system. Based on these findings, the three strains and their microbial consortium show promise for application in biofertilizers, to improve soil health and facilitate optimal plant growth.

## Introduction

Agriculture is a significant contributor to the economy of Viet Nam, accounting for approximately one-fifth of the country’s gross domestic product, and plays an important role in the labor force and income source for the country’s population [1, 2]. Thus, the use of chemical pesticides has increased rapidly in Vietnam to enhance agricultural production [3]. From 1991 to 2010, the sharp increase in the variety of pesticide-active ingredients reflected the growing reliance on chemical pesticides in agricultural practices, with the number increasing from 77 to 437 types of pesticide-active ingredients [4]. Unless these chemical pesticides are completely degraded, they will bind to soil particles and persist in all environmental compartments [5, 6]. The lack of degradation can lead to bioaccumulation, and these fungicides may accumulate in higher concentrations as they pass through various organisms in the food chain [7, 8]. The widespread use of pesticides raises concerns about persistent organic pollutants [9], and their potential adverse impacts on soil biodiversity and human health [5, 10, 11].

Triazole fungicides (TFs) are systemic pesticides that are widely used to prevent fungal diseases in vegetables, fruits, and crops, especially in Viet Nam [4, 8, 12]. However, several studies have suggested that residual TFs have the most significant effect on soil microbial communities and plant health in general [8, 13-15]. Three widely used TFs, hexaconazole, propiconazole and difenoconazole have been found to adversely affect plant metabolic activities, causing serious effects on plant growth and development [16-19]. It is crucial, therefore, to develop effective methods to remove residual TFs from the soil, to preserve the balance and functionality of the soil ecosystem and ensure long-term environmental sustainability in agriculture and beyond [20, 21].

Bioremediation is a method of applying microorganisms or enzymes to restore an environment contaminated with fungicides [22]. Recently, bioremediation techniques have gained significant attention due to their environmentally friendly approach to reducing toxic chemicals in contaminated soils [22-24]. In recent advances, bioremediation has been presented as a cost-effective and time-efficient solution, with the potential to replace traditional remediation methods [25]. Several bacterial genera are known to have the capacity to degrade pesticides in soil and promote plant growth [16, 26-31]. These functionalities contribute to promoting plant growth and soil health, making them valuable assets for improving agricultural productivity and sustainability. However, effective biodegradation with these microbes depends on specific environmental conditions, and completing the degradation of pollutants using a single strain is challenging [32, 33]. Pesticides can be complex and persistent by accumulation in the long term, and relying on a single microbial strain may not provide sufficient metabolic diversity or enzymatic capacity to fully degrade them. The co-culturing of microbial consortiums or combinations of different strains can increase the adaptability of the consortiums to varying environmental conditions and enhance their efficacy in degrading a wide range of pollutants, proving to be more effective than a single-strain approach [21, 24, 29, 34]. For example, co-cultures of two bacterial isolates have been reported to significantly improve fungicide degradation in vitro [33] or in field soils [35].

Hence, the primary objective of the present study was to assess the biodegradation potential of bacterial strains that have been isolated from agriculturally contaminated soil. This assessment is focused on their capacity to biodegrade three extensively utilized TFs, namely hexaconazole, propiconazole, and difenoconazole under controlled laboratory conditions. Beyond this fundamental examination, this study also examined the role of a bacterial mixed culture, exploring its ability to not only facilitate the breakdown of these aforementioned fungicides but also promote plant growth. This research is driven by the pressing need to understand and harness the potential of these soil bacteria for remediation purposes and sustainable agricultural practices, and ultimately to reduce the environmental footprint of fungicide use in farming, thereby contributing to the broader objective of ensuring a healthier and more productive environment.

## Materials and Methods

### Enrichment Culture, Isolation, and Identification of TF-Degrading Strains

Soil samples were taken from vegetable- and flower-growing areas in Da Lat, where fungicides are frequently used during cultivation. Detailed information on geographic coordinates and residues of fungicides in the soil can be found in Table S1. The soil samples from the the lower layer by depths of 0-10 cm were collected, transported to the laboratory, and used for inoculation. For microbial enrichment and cultivation, mineral salt medium (MSM) was used [36]. Three TFs, hexaconazole, propiconazole, and difenoconazole (Sigma-Aldrich, Germany), were supplemented in MSM medium at a concentration of 100 mg/l of each TF. After thorough mixing, 10 g of soil was added to a 250-ml glass bottle containing 90 ml of MSM, and the culture was incubated in the dark at room temperature in a rotating shaker (110 rpm). When the medium became cloudy, the culture was transferred to a fresh-growth medium. After five sub-culture steps, the serial dilutions of the culture were spread on MSM agar plates containing TFs (100 mg/l of each TF) and incubated at 30°C. After 3 days of culture, discrete and morphologically different colonies were formed to create pure strains by streaking on tryptone soya agar (TSA, Sigma-Aldrich, Germany). The isolates were streaked on fresh TSA agar plates and maintained on both agar and liquid TSA media.

To determine the TFs’ tolerance potential, the bacterial strains were grown in MSM agar plates containing TFs, as the sole carbon and energy source, at concentrations of 200 mg/l, 300 mg/l, 400 mg/l, and 500 mg/l of each TF. The TF-degrading isolates were confirmed by determining the residual concentration of TFs in the liquid MSM by gas chromatography GC-2010 Plus (Shimadzu, Japan). Briefly, the cultures were prepared by adding 1 ml of 10^6^ CFU/ml inoculum to 99 ml of liquid MSM supplemented with TFs at concentrations of 100 mg/l. The TF-containing liquid MSM without inoculum was used as a negative control. The detection of residual TFs was performed with an interval of 3 days after growth. Additionally, bacterial strains were tested for their compatibility with the cross-streak method, and no lysis was found at the juncture. Three isolates with the highest tolerance and TF degradation potential, and no antagonistic interactions, designated as D5-2, D9-1, and D10-3, were selected for further study.

The selected strains were identified by sequencing the 16S rRNA gene fragment with the universal forward and reverse primers 27F (5'-AGAGTTTGATCCTGGCTCAG-3') and 1492R (5'-GGTTACCTTGTTACGACTT-3')[37]. The total genomic DNA of these strains was extracted by Bacteria DNA Preparation (Jena Bioscience, Germany). The PCR mixtures of 50 μl contained 2 μl of DNA, 1.5 μl of each primer, 5 μl EZ PCR mix (PhuSa Genomics, Vietnam), and 40 μl H_2_O. The thermal cycling parameters were 1 min at 95°C for initial denaturation, 35 cycles of 30 s at 95°C, 30 s at 55°C, 90 s at 72°C, and a final extension at 72°C for 5 min. PCR products were visualized on 2% agarose gels and purified by PCR Purification Kit (Jena Bioscience, Germany). The PCR products were analyzed by ABI3500 Genetic Analyzer (Thermo Fisher Scientific, USA). Sequence reads were identified in the GenBank database (NCBI, USA) using standard nucleotide BLAST searches. The 16S rRNA gene sequences of the three strains, D5-2, D9-1, and D10-3, were deposited into the GenBank database under accession numbers OQ154875, OP630837, and OQ154876, respectively.

### Determination of the Auxiliary Characteristics of Selected Bacterial Isolates Free Nitrogen Fixation

The ability of each bacterial isolate to fix free nitrogen was tested by growing the strains in 99 ml of Ashby's aqueous medium [38], which was inoculated with 1 ml of cell suspension (OD_600_ = 0.7) and incubated in shaking conditions at 120 rpm and 30°C for 4 days. The culture was collected and centrifuged by centrifugation (13,000 rpm) for 2 min. The colorimetric method with Nessler’s reagent [39] was used to determine the content of ammonia formed in the supernatant.

### Indole Acetic Acid Production

The IAA production of the three strains in culture broth was determined using the colorimetric method by the improved Salkowski method [40]. All isolates were grown in King's medium B (Merck, Germany) at 30°C for 24 h. The culture was centrifuged at 10,000 ×*g* for 15 min. Subsequently, 1 mL of the supernatant was mixed with 2 ml of Salkowski’s reagent in Durham tubes and incubated in the dark at 30°C, for 10 min for a complete reaction. The absorbance was measured at 530 nm. The concentration of IAA produced by the bacterial strains was determined using a standard curve of IAA [41].

### Phosphate Solubilization

Phosphate solubilization was carried out on the Pikovskaya agar medium by determining the formation of light rings around the colonies of the strain-scoring cultures according to the method described previously [42]. The presence of a transparent area around the bacterial colonies after one week of incubation at 30°C indicates phosphorus solubilization. The phosphorus solubilization efficiency is measured by computing the Solubilization Index (SI), which is SI = (colony diameter + halo zone diameter)/colony diameter.

### Cellulose Degradation

The preliminary screening for cellulose degradation was tested by streaking bacterial isolates in Congo red cellulose agar media, as described in a previous study [43]. The cellulose degradation activity of bacterial strains was analyzed by measuring the Congo red discoloration zone around the bacterial colony versus the bacterial colony in millimeters [44]. Hydrolytic capacity (HC) was estimated by the ratio of clearance zone to bacterial colony diameter [45].

### Plant-Microbe Interaction and Degradation of TFs in Pot Experiment

The pot experiment was carried out with 15-day-old Lollo Rossa lettuce (*Lactuca sativa* L.), in the greenhouse of the Nuclear Research Institute, Da Lat, Viet Nam (1157'23.9"N, 10827'12.9"E). The natural soil was collected and sieved with a 2-mm sieve to remove weeds and stones, and then placed in a greenhouse maintained at a temperature range of 23–27°C and air humidity of 73–88%. Soil samples before experimenting were analyzed for physicochemical properties, such as pesticide residues, pH, total nitrogen, total phosphorus, total potassium, humus content, and the total number of aerobic bacteria (Table S2).

The chemical fungicides selected for the experiment were commercially known as Tilt Super 300EC (150 g/l each of difenoconazole and propiconazole) (Syngenta, Switzerland), and Anvil 5SC (50 g/l hexaconazole)(Syngenta, Switzerland). The experiment was performed by arranging four treatments, each with three replications, in a trial pot area of 1.0 m² (1 × 1 m), with a plant density of 20 plants per pot. The experiment was conducted for 45 days after planting (DAP). The experimental conditions for each treatment are presented in Table 1. In brief, a mixture of fungicides was spiked once into the soil one day before planting with a concentration of 50 mg/kg of each TF, respectively. The inoculum (10^6^ CFU/ml) of selected strains was mixed at a ratio of 1:1:1 and added to the soil six times continuously with a concentration of 20 ml/5 L of water. The initial application occurred immediately after planting, followed by subsequent applications at 7-day intervals. Plant growth parameters such as survival rate (%), plant height (cm), and fresh weight (g) were recorded at 45 DAP. Analysis of pesticide content in soil used GC-2020 Plus (Shimadzu, Japan) using a method similar to that of previously published papers [46, 47]. In brief, 25 g of soil samples were weighed into 250 ml conical flasks, with 100 ml of dichloromethane added. The mixture was shaken for 3 s and then placed in an ultrasonic bath for 30 min. The extract was filtered through a filter paper (Whatman No. 4) containing sodium sulfate and transferred to a beaker. After that, 50 ml of the filtrate was dried out to get 10 ml. The extract was then transferred to a graduated micro-vial and dried with nitrogen gas. The residue was dissolved with 1 ml of acetone, mixed in a vortex mixer, and filtered through a 0.45-μm filter before injection into the GC-ECD. Bacterial cell density and residual fungicide concentration in the soil were monitored at 0, 7, 21, 35, and 45 DAP.

### Data Analysis

The data were subjected to statistical analyses to calculate the mean, and standard deviation (SD). The significance (*p* < 0.05) of differences was analyzed using two-way ANOVA and assessed by post-hoc comparison of means. All statistical analyses were conducted using SPSS 25.0 software.

## Results and Discussion

### Isolation and Characterization of TF-Degrading Strains

From the enrichment culture, several promising morphologically different colonies were isolated and purified. All purified isolates were grown in MSM supplemented with a concentration of 300 mg/l TF (100 mg/l for each TF). The three isolates, namely D5-2, D9-1, and D10-3, demonstrated the ability to grow in the supplemented medium with TF concentrations of up to 1,500 mg/l (500 mg/l for each TF) indicating their tolerance to higher levels of TFs in the medium. The colonies of bacterial strains D5-2, D9-1, and D10-3 exhibited distinct characteristics (Fig. S1). Strain D5-2 showed round, milky-white colonies. Strain D9-1 exhibited round white colonies, while strain D10-3 displayed flat colonies with a yellow hue. The ability of soil-inhabiting microorganisms to withstand pesticides is a promising feature. Microbes capable of tolerating high doses of fungicide are likely to also be involved in fungicide degradation, which is an important trait influenced by their unique physiological and genetic characteristics. The screening results suggested the potential of the three isolates for further investigation in understanding TF degradation or utilization processes. Several soil bacteria have been reported, exhibiting the dual ability to both tolerate and degrade pesticides, and even promote plant growth [16, 30, 31].

To identify selected isolates, sequences of the 16S rDNA gene were obtained and analyzed through BLASTn against the GenBank database. Based on 16S rRNA gene analysis, these isolates were identified as *Klebsiella* sp., *Citrobacter* sp., and *Pseudomonas* sp. The 16S rDNA sequence of isolate D5-2 was closely aligned with undescribed taxonomic strains CY48 (ON386909), OX_M (KF86467), and *Klebsiella oxytoca* (KT767973, KT767972, KM349413, and KC155255) with 99.93% similarity, respectively. On the contrary, isolate D9-1 showed a higher 16S rRNA similarity of 99.64% to *Citrobacter freundii* UMH16 (CP024677), while isolate D10-3 exhibited a 100%similarity to *Pseudomonas aeruginosa* strains. From our enrichment culture, several strains belonging to different taxa were isolated, indicating that multiple genera can tolerate fungicides and may participate in the TF degradation process. Several of the isolates capable of withstanding high levels of fungicide are reported to belong to the genera *Klebsiella* [27, 48, 49], *Citrobacter* [28, 50], and *Pseudomonas* [29, 30].

### Bacterial Growth and Biodegradation of TFs

**Fig. 1:**
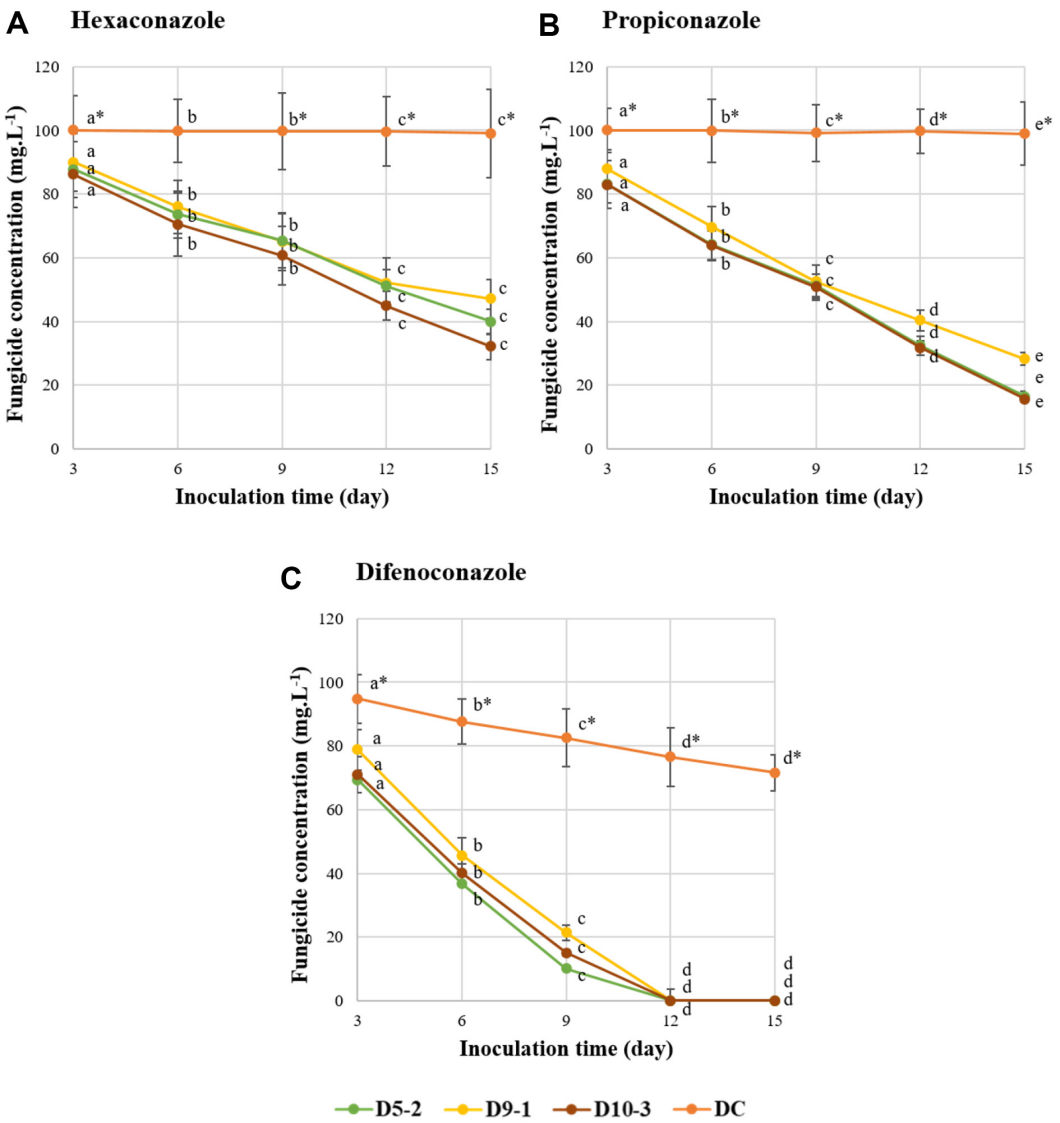
Degradation of three TFs compounds: hexaconazole (A), propiconazole (B) and difenoconazole (C), during the growth of three bacterial strains (D5-2, D9-1, and D10-3). Negative controls (DC) lacked bacterial strains in the culture medium. Different letters indicate statistically significant differences (*p* < 0.05). The error bars represent the SD and the values are the means of three replicates.

The selected strains were able to utilize TFs as the sole carbon and energy source with partially or completely decomposed TFs after 15 days of incubation (Fig. 1). The degradation of TFs by bacterial strains was exhibited as a decrease in concentration, with different rates being observed depending on the TF and bacterial strain (ANOVA, *p* < 0.05). After 15 days, as in Fig. 1, strain D5-2 degraded 60.06% and 83.51% of hexaconazole and propiconazole, while strain D9-1 degraded 52.86% and 71.77%, and strain D10-3 degraded 67.87% and 84.35%, respectively, compared to the negative control, with losses of less than 1.1% of the initial concentration. We noticed that the difenoconazole was completely decomposed after 12 days. The three strains degraded TFs (hexaconazole, propiconazole and difenoconazole) at different rates: D5-2 (4.00, 5.56, 9.98 mg/l/d), D9-1 (3.52, 4.78, 8.73 mg/l/d), and D10-3 (4.52, 5.62, 9.44 mg/l/d). However, the degradation efficiency of the three strains was not significantly different (two-way ANOVA, *p* > 0.05). Several TF-degrading microorganisms have been reported to belong to different species, each exhibiting varying degradation rates. For instance, *Sphingobacterium multivorum* B-3 decomposed 85.6% of the hexaconazole in a liquid culture medium with a concentration of 50 mg/L within 6 days [51]. In other studies, *Ensifer* sp. B2 degraded difenoconazole by up to 85% in a culture medium with a concentration of 100 mg/l within just 24 h of incubation [52]. Similarly, *Lysinibacillus* sp. BTKU3 also exhibited the ability to degrade difenoconazole at a concentration of 9.1 μg/ml within 3 days [53]. Meanwhile, *Burkholderia* sp. BBK_9 exhibited the degradation of propiconazole at 8.89 μg/ml within a 4-day timeframe under optimal conditions [54]. This observation indicates that the TF degradation effectiveness is influenced by both the nature of the TFs and the unique characteristics of the bacterial strains.

### Determination of Auxiliary Characteristics

The quantitative evaluation of selected isolates for plant growth-promoting activities was determined (Table 2). Among the three isolates, D5-2 showed high nitrogen fixing (4.80 ± 0.38 mg/l) and cellulose degradation (10.2 ± 0.71 mm) compared with D10-3 (1.57 ± 0.11 mg/l and 8.4 ± 0.76 mm) and D9-1 (1.04 ± 0.10 mg/l and 7.7 ± 0.62 mm), respectively. In contrast, the phosphorus solubilization and IAA production of three strains showed minor differences in D5-2 (13.5 ± 0.81 mm and 9.12 ± 0.73 mg/l), D10-3 (11.3 ± 1.13 mm and 11.68 ± 1.17 mg/l), and D9-1 (15.8 ± 1.42 mm and 10.24 ± 0.92 mg/l). The nitrogen-fixing and phosphate-solubilizing properties of the three isolates are recognized as important features in the agricultural soil system. Their ability to convert atmospheric nitrogen into ammonia [26, 30, 31], as well as insoluble inorganic phosphate compounds into available forms [26, 55, 56], plays a vital role in providing essential nutrients that living organisms can use for biosynthesis. Additionally, during the growth of our strains, cellulose degradation was also observed, facilitating the conversion of organic waste into valuable organic matter and contributing to the reduction of the C:N ratio while also enhancing soil productivity [57, 58]. The ability of the three isolates to produce IAA, a crucial growth-augmenting phytohormone, becomes especially significant for survival in harsh environments. This allows plants to access the growth-promoting benefits of IAA, even under conditions of low-nutrient soils or elevated levels of fungicide stress [26, 42, 59].

### Soil Bioremediation and Enhancement of Plant Growth

**Fig. 2:**
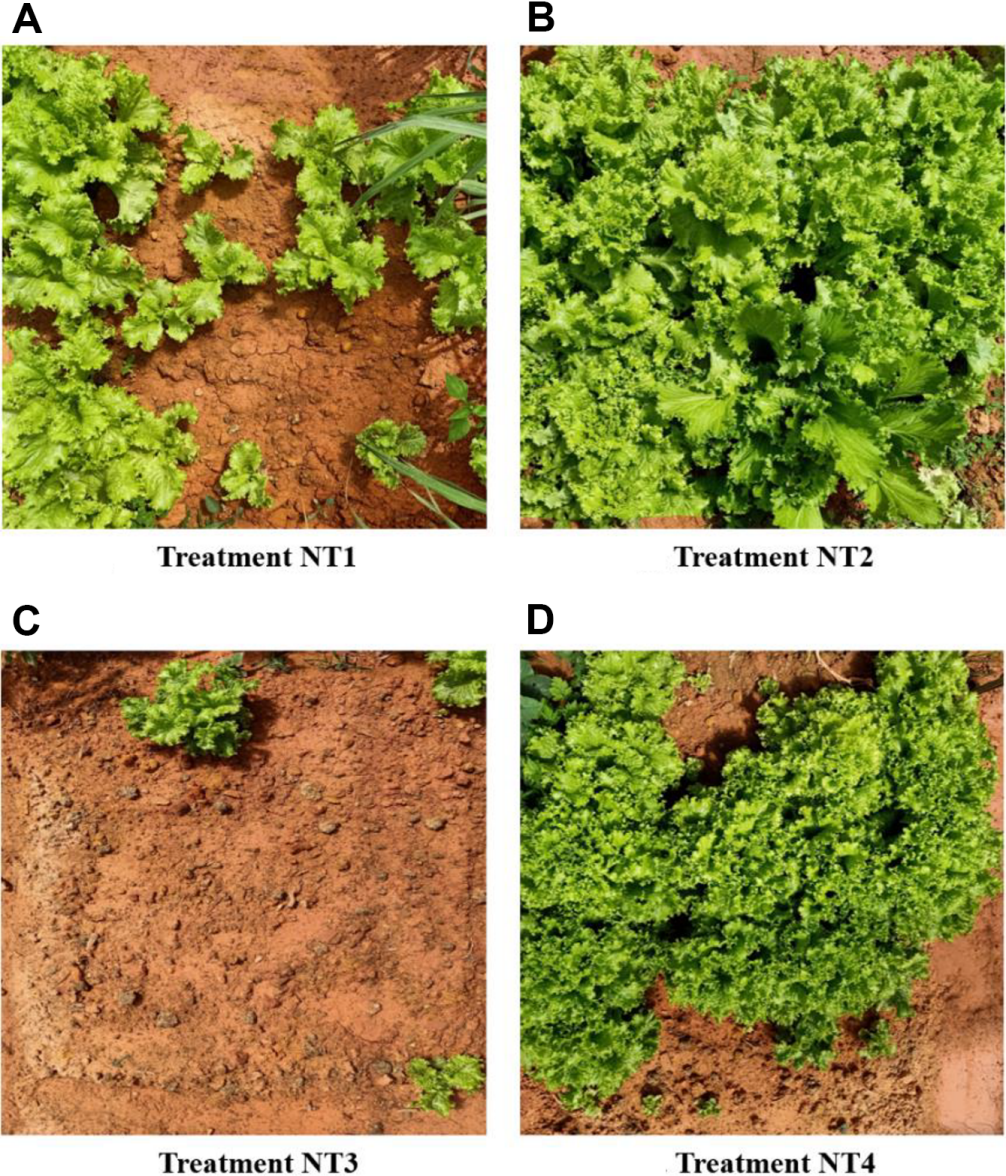
Growth experiment with Green Lollo Rossa lettuce in soil supplemented with bacterial mixtures and TFs (150 mg/kg) after 45 DAP. Treatments: (**A**) NT1 without TFs and bacterial mixtures, (**B**) NT2 with bacterial mixtures, (**C**) NT3 with TFs, and (**D**) NT4 with both TFs and bacterial mixtures.

The effects on plant growth parameters and TF biodegradation of the three-isolate mixtures and control in pot experiments were determined in Lollo Rosso lettuce, in a natural soil without or with bioaugmentation treatment microorganisms (NT1 and NT2), and in the presence of fungicide pressure (NT3 and NT4). Plants grown in soil with a bacterial supplement showed a significant improvement in growth, both in terms of height and fresh weight (ANOVA, *p* < 0.05). At a similar survival rate in NT1 and NT2, bacterial addition to soil showed increases in plant height (from 9.9 ± 1.5 to 22.3 ± 1.5 cm) and fresh weight (from 46 ± 11.5 to 135.8 ± 7.2 g) (Table 3, Fig. 2A and 2B). Bacterial density in the soil samples from treatment NT2 was higher than that from treatment NT1 with a significant difference (Fig. S2, ANOVA, *p* < 0.05). As shown in Table S3, the initial soil is poor in nutrients, in terms of nitrogen, potassium, and humus content. The addition of a bacterial mixture helped to improve the growth and development of plants; at the same time, an increase in the bacterial abundance of the soil was also observed. In this study, three bacterial strains have confirmed their ability to enhance plant growth. The genera to which they belong are recognized for their role in promoting plant growth in the rhizosphere [60-62]. These rhizosphere bacteria possess the ability to colonize plant roots and provide various benefits to their host plants, acting as biofertilizers. They improve availability by fixing atmospheric nitrogen and solubilizing soil minerals, such as phosphorus and potassium. In addition, they increase the production of plant hormones, such as auxins, cytokinins, abscisic acid, and gibberellins, while reducing ethylene levels [16, 60].

In contrast, the addition of TFs to the soil had a pronounced detrimental effect on plants, as in the NT3 treatment. This led to a significant decrease in survival rate, with a notable reduction of 18.3 ± 7.6% compared to 98.3 ± 2.8% in the control treatment (Table 3, Fig. 2C). Both other plant parameters and total bacterial density exhibited a reduction. In NT4 treatment, the addition of three-isolate mixtures to the TF-supplemented soil showed an improvement in a survival rate of 75.0 ± 5.0%, as well as plant height (18.3 ± 0.9 cm) and fresh weight (115.5 ± 15.7 g). Despite the presence of TF pressure, the total bacterial density was observed to be higher in treatment NT4 as compared to NT3, and even higher than in control treatment NT1 (Fig. S2). In earlier studies, the authors observed that the widespread use of TFs in agricultural soils has a negative impact on soil microbiota [8, 63], both in terms of abundance and diversity, and by reducing the activity of many enzymes. This finding confirmed that supplementation of TF-degrading strains in the soil can mitigate the toxic effects of TFs while simultaneously enhancing the growth of plants.

**Fig. 3:**
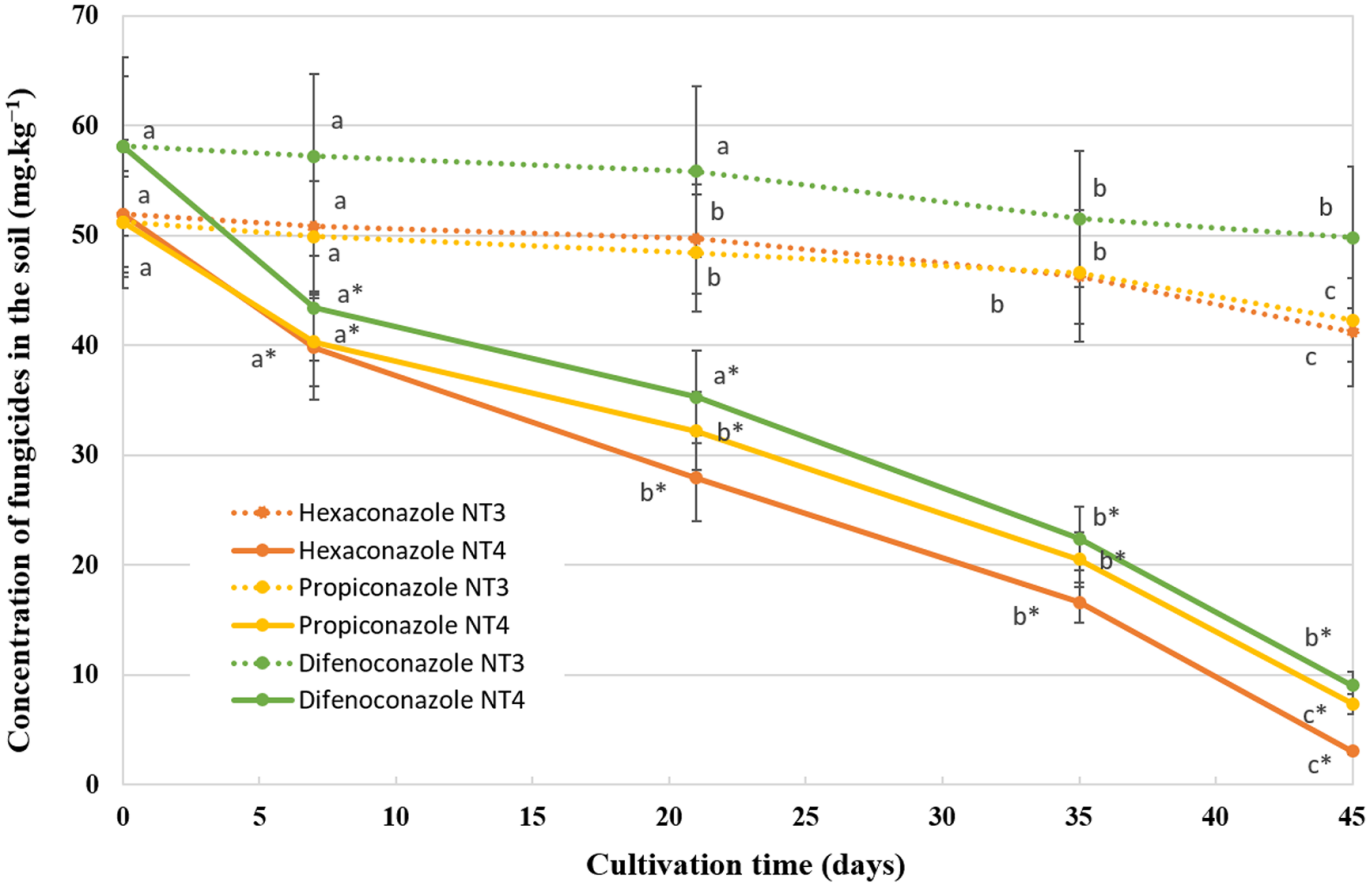
Degradation of TFs in treatment NT3 and NT4 with an initial total TF concentration of 150 mg/kg soil, respectively. Values are the means of three replicates, and error bars represent SD. Different letters indicate statistically significant differences (*p* < 0.05).

Gas chromatography was used to confirm the reduction of TFs in NT3 and NT4 treatments (Fig. 3). In these treatments, TFs were added to the soil at a concentration of 50 mg/kg (51.9 mg/kg hexaconazole; 51.2 mg/kg propiconazole, and 58.1 mg/kg difenoconazole). Although the concentrations of hexaconazole, propiconazole, and difenoconazole are 10 times higher than prescribed, significant reductions of 94.12, 85.64, and 84.46% were observed in NT4 treatment, respectively. In contrast, approximately 80% of the applied TFs persisted until the conclusion of the cultivation period in NT3 treatment. The TF degradation revealed significant differences between different treatments (ANOVA, *p* < 0.05). These results suggest that the TF-degrading activities of the three isolates are displayed in the soil-plant system in the form of a consortium. Several reports have focused on the isolation and characterization of TF-degrading microbes and microbial communities [15, 16, 31, 35, 49, 64]. The metabolic pathways of TFs and the fate of several metabolites have been reported in studies involving *Pseudomonas* [65], *Klebsiella* [66], and *Citrobacter* [67]. However, there are a limited number of studies on the degradation pathway of TFs in microorganisms. The previous experiment showed that cytochrome P450 monooxygenase was mainly involved in the propiconazole degradation of *P. aeruginosa* [65]. The identification of different metabolites allowed the formulation of a shared metabolic pathway for pesticide degradation within certain bacterial species [68, 69]. However, the promotion of TF biodegradation in these organisms can occur through varied metabolic processes [69]. In several studies, microbial co-cultures or consortiums of various genera possess the ability to tolerate pesticides and are actively involved in the pesticide degradation process [21, 35, 62].

## Conclusion

The present study may provide the foundation for the development and utilization of TF-degrading bacterial strains in agricultural plant-soil systems. Three bacterial strains, namely D5-2, D9-1, and D10-3, with the potential to tolerate and degrade TFs, were obtained. The strains investigated here exhibited several important characteristics, such as biological nitrogen fixation, phosphate solubilization, IAA production, and cellulose degradation, which can increase the availability of nutrient concentrations in the rhizosphere. Significant improvement was observed in lettuce plants treated with the application of bioaugmentation in the soils. Nevertheless, the current study has certain limitations involving the lack of microbial genome information on the isolates and an evaluation of their subsequent impact on indigenous bacterial communities in the soil and plant roots. Subsequent research endeavors should aim to comprehensively characterize the genomic features and metabolism of the TF-degrading microorganisms isolated in this study. Additionally, further studies are required to investigate the relationship among isolates and resident bacterial communities in soil and plant roots for effective remediation and mitigation of TF contamination.

## Supplemental Materials

Supplementary data for this paper are available on-line only at http://jmb.or.kr.



## Figures and Tables

**Table 1:** Summary of experimental conditions for each set of trial pots.

Treatment	Apply fungicide		Bacterial mixed culture
	Anvil 5SC	Tilt Super 300EC	
NT1	-	-	-
NT2	-	-	+
NT3	+	+	-
NT4	+	+	+

**Table 2:** Plant growth-promoting characteristics of three isolates.

Bacterial strain	NH4+ (mgl^-1^)	IAA (mgl^-1^)	Phosphate SI (mm)	Cellulose HC (mm)
D5-2	4.80 ± 0.38^a^	9.12 ± 0.73^a^	13.5 ± 0.81^a^	10.5 ± 0.71^a^
D9-1	1.04 ± 0.10^b^	10.24 ± 0.92^a^	15.8 ± 1.42^a^	7.7 ± 0.62^a^
D10-3	1.57 ± 0.11^b^	11.68 ± 1.17^a^	11.3 ± 1.13^a^	8.4 ± 0.76^a^

IAA = indole acetic acid; SI = solubilization index; HC = hydrolytic capacity. The values indicate the mean ± SD of three replicates. The means in the same column followed by the different letters are significantly different at *p* < 0.05.

**Table 3:** Effects of plant growth parameters and TF biodegradation of the three isolates and the control in pot experiment on Lollo Rosso lettuce.

Growth parameters	Treatment NT1	Treatment NT2	Treatment NT3	Treatment NT4
Survival rate (%)	98.3 ± 2.8^a^	98.3 ± 2.9^a^	18.3 ± 7.6^b^	75,0 ± 5.0^c^
Height (cm)	9.9 ± 1.5^a^	22.3 ± 1.5^b^	9.2 ± 0.9^a^	18.3 ± 0.9^c^
Fresh weight (g)	46.0 ± 11.5^a^	135.8 ± 7.2^b^	32.3 ± 9.2^a^	115.5 ± 15.7^b^

The values indicate the mean ± SD of three replicates. The means in the same row followed by different letters are significantly different at *p* < 0.05.
