# Supplementary material for: Degradation of Triazole Fungicides by Plant Growth-Promoting Bacteria from Contaminated Agricultural Soil
Source: J Microbiol Biotechnol. 2023 Oct 26;34(1):56–64. doi: 10.4014/jmb.2308.08037 (PMC10840487; doi:10.4014/jmb.2308.08037)
Supplement: Supplementary file 1 [file jmb-34-1-56-supple.pdf]

J. Microbiol. Biotechnol.
https://doi.org/10.4014/jmb.2308.08037

Supplementary Tables and Figures

Fungicide degradation by plant growth-promoting bacteria from contaminated agricultural soils

Table S1. List of soil samples used for the enrichment of microorganisms in this study with their characteristics.

Name	Location (GPS)	Type of crops	Time of using pesticides (years)	Residues of fungicides in the soil (mg.kg^{-1})		
				Hexaconazole	Propiconazole	Difenoconazole
D1	11.967409 N, 108.454337 E	Chrysanthemum	10	0.23 ± 0.17	0.02 ± 0.14	0.38 ± 0.21
D2	11.966227 N, 108.457984 E	Potato	7	0.017 ± 0.02	0.034 ± 0.03	0.022 ± 0.03
D3	11.993736 N, 108.428945 E	Cabbage	10	0.045 ± 0.07	0.45 ± 0.15	0.048 ± 0.04
D4	11.977864 N, 108.432624 E	Onion paro	8	0.037 ± 0.04	0.037 ± 0.03	0.032 ± 0.04
D5	11.949805 N, 108.404361 E	Rose	10	0.54 ± 0.15	0.97 ± 0.14	0.69 ± 0.17
D6	11.949972 N, 108.404305 E	Lily	5	0.34 ± 0.19	0.36 ± 0.11	0.68 ± 0.13
D7	11.945464 N, 108.483962 E	Lily	10	0.22 ± 0.18	0.76 ± 0.16	0.48 ± 0.18
D8	11.945264 N, 108.483972 E	Lettuce	8	0.036 ± 0.05	0.026 ± 0.02	0.072 ± 0.06
D9	11.962192 N, 108.475422 E	Chrysanthemum	9	0.37 ± 0.16	0.43 ± 0.14	0.79 ± 0.18
D10	11.962194 N, 108.475444 E	Cabbage	6	0.027 ± 0.03	0.053 ± 0.05	0.041 ± 0.05

Table S2. Physico-chemical characteristics of soil samples used for pots experiment. Abbreviations: ND - not detected, LOQ - limit of quantification.

No	Parameter	Unit	Results
1	pH	-	6.73 ± 0.02
2	Total nitrogen	%	0.92 ± 0.07
3	Total phosphorus	%	0.38 ± 0.03
4	Total potassium	%	0.14 ± 0.01
5	Total humus	%	0.86 ± 0.05
6	Total aerobic bacteria	$CFU.g^{-1}$	$3.8x10^3$ ± 572
7	Fipronil	$mg.kg^{-1}$	ND (LOQ=0.002)
8	Bifethrin	$mg.kg^{-1}$	ND (LOQ=0.01)
9	Cyfluthrin	$mg.kg^{-1}$	ND (LOQ=0.01)
10	Cypermethrin	$mg.kg^{-1}$	ND (LOQ=0.01)
11	Deltamethrin	$mg.kg^{-1}$	ND (LOQ=0.01)
12	Fenpropathrin	$mg.kg^{-1}$	ND (LOQ=0.01)
12	Lambda cyhalothrin	$mg.kg^{-1}$	ND (LOQ=0.01)
14	Permethrin	$mg.kg^{-1}$	ND (LOQ=0.01)
15	Chlorpyrifos ethyl	$mg.kg^{-1}$	ND (LOQ=0.01)
16	Chlorpyrifos methyl	$mg.kg^{-1}$	ND (LOQ=0.01)
17	Diazinon	$mg.kg^{-1}$	ND (LOQ=0.01)
18	Profenofos	$mg.kg^{-1}$	ND (LOQ=0.01)
19	Endosulfan	$mg.kg^{-1}$	ND (LOQ=0.01)
20	Propiconazole	$mg.kg^{-1}$	ND (LOQ=0.01)
21	Difenoconazole	$mg.kg^{-1}$	ND (LOQ=0.01)
22	Azoxystrobin	$mg.kg^{-1}$	ND (LOQ=0.01)
23	Cyantraniliprole	$mg.kg^{-1}$	ND (LOQ=0.01)
24	Indoxacarb	$mg.kg^{-1}$	ND (LOQ=0.01)
25	Kresoxim methyl	$mg.kg^{-1}$	ND (LOQ=0.01)
26	Chlorothalonil	$mg.kg^{-1}$	ND (LOQ=0.01)
27	Triadimefon	$mg.kg^{-1}$	ND (LOQ=0.01)
28	Trifloxystrobin	$mg.kg^{-1}$	ND (LOQ=0.01)

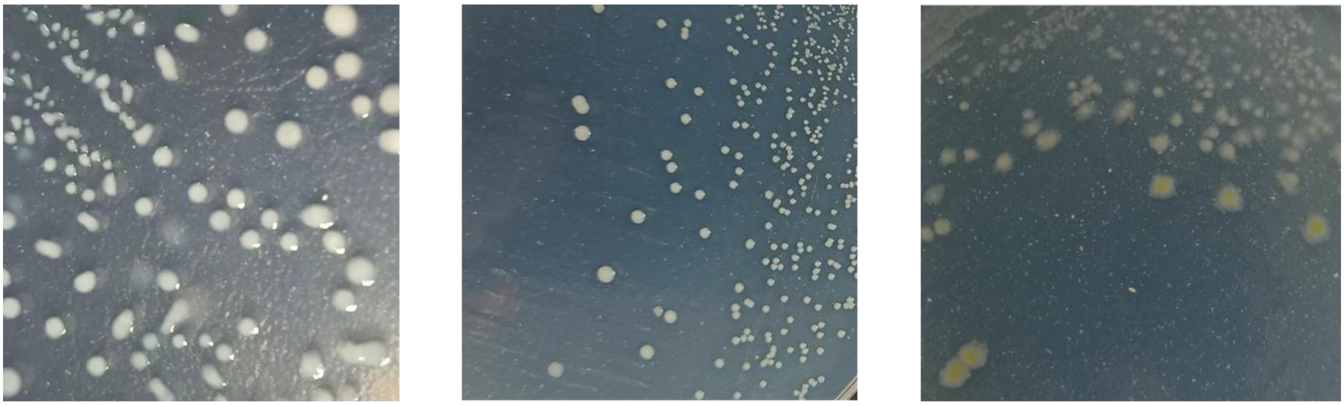

A B C

Fig. S1. Colony morphology of three bacteria strains were grown on TSA medium D5-2 (A), D9-1 (B), D10-3 (C).

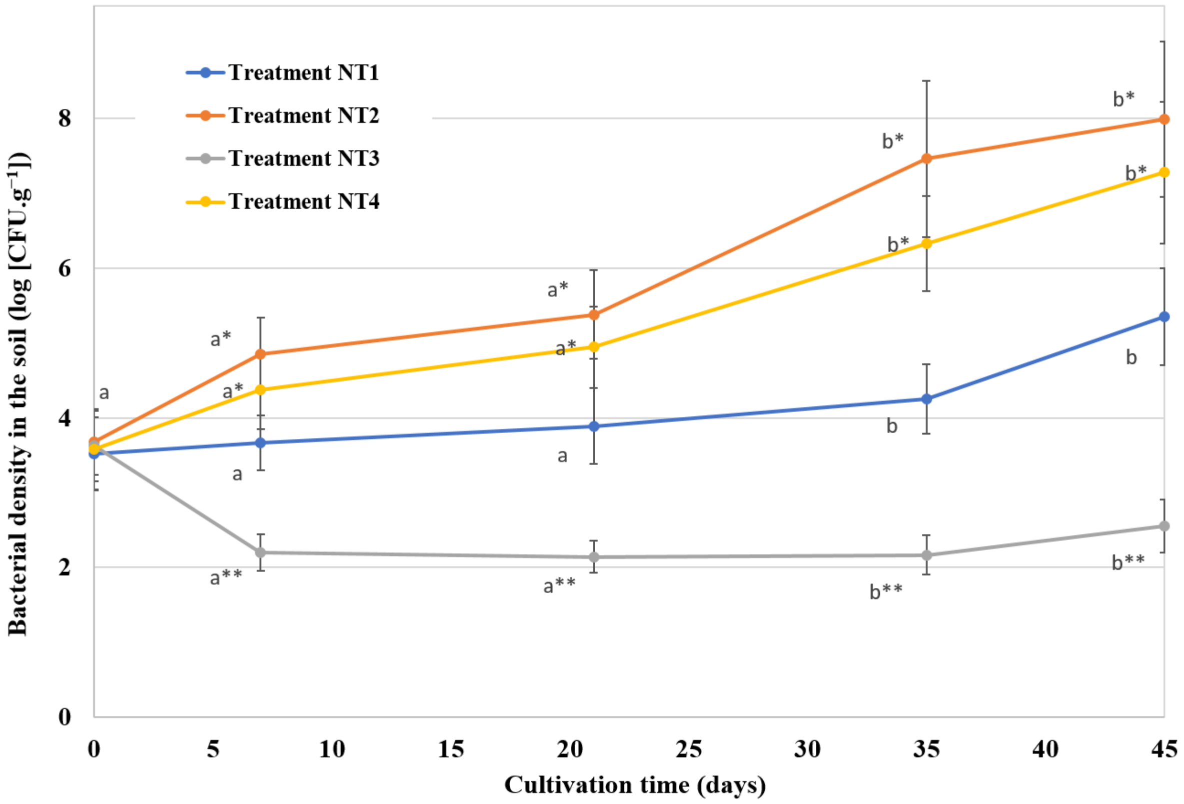

Fig. S2. Total bacterial density in the soil in 4 treatments (NT1, NT2, NT3, and NT4). Values are the means of three replicates and error bars represent SD. Different letters indicate statistically significant differences ($p<0.05$).
